# Anemoside B4 Inhibits Vascular Smooth Muscle Cell Proliferation, Migration, and Neointimal Hyperplasia

**DOI:** 10.3389/fcvm.2022.907490

**Published:** 2022-05-10

**Authors:** Dan Shan, Ping Qu, Chao Zhong, Luling He, Qingshan Zhang, Guoyue Zhong, Wenhui Hu, Yulin Feng, Shilin Yang, Xiao-feng Yang, Jun Yu

**Affiliations:** ^1^Center for Translational Medicine, Jiangxi University of Traditional Chinese Medicine, Nanchang, China; ^2^Department of Cardiovascular Sciences and Center for Metabolic Disease Research, Lewis Katz School of Medicine, Temple University, Philadelphia, PA, United States; ^3^Department of Internal Medicine, Affiliated Hospital of Inner Mongolia University for the Nationalities, Tongliao, China; ^4^Research Center of Natural Resources of Chinese Medicinal Materials and Ethnic Medicine, Jiangxi University of Traditional Chinese Medicine, Nanchang, China; ^5^The National Pharmaceutical Engineering Center for Solid Preparation in Chinese Herbal Medicine, Nanchang, China

**Keywords:** Anemoside B4, vascular smooth muscle cell, proliferation, migration, neointimal hyperplasia

## Abstract

Vascular smooth muscle cell (VSMC) phenotypic transformation, proliferation, and migration play a pivotal role in developing neointimal hyperplasia after vascular injury, including percutaneous transluminal angioplasty and other cardiovascular interventions. Anemoside B4 (B4) is a unique saponin identified from the Pulsatilla chinensis (Bge.) Regel, which has known anti-inflammatory activities. However, its role in modulating VSMC functions and neointima formation has not been evaluated. Herein, we demonstrate that B4 administration had a potent therapeutic effect in reducing neointima formation in a preclinical mouse femoral artery endothelium denudation model. Bromodeoxyuridine incorporation study showed that B4 attenuated neointimal VSMC proliferation *in vivo*. Consistent with the *in vivo* findings, B4 attenuated PDGF-BB-induced mouse VSMC proliferation and migration *in vitro*. Moreover, quantitative RT-PCR and Western blot analysis demonstrated that B4 suppressed PDGF-BB-induced reduction of SM22α, SMA, and Calponin, suggesting that B4 inhibited the transformation of VSMCs from contractile to the synthetic phenotype. Mechanistically, our data showed B4 dose-dependently inhibited the activation of the phosphatidylinositol 3-kinase (PI3K)/AKT and p38 mitogen-activated protein kinase MAPK signaling pathways. Subsequently, we determined that B4 attenuated VSMC proliferation and migration in a p38 MAPK and AKT dependent manner using pharmacological inhibitors. Taken together, this study identified, for the first time, Anemoside B4 as a potential therapeutic agent in regulating VSMC plasticity and combating restenosis after the vascular intervention.

## Introduction

Vascular smooth muscle cell (VSMC) is the major cell type in arteries. Unlike terminally differentiated cardiac or skeletal myocytes, VSMCs retain remarkable plasticity (1). VSMCs can be modulated from a quiescent status to a hyperproliferative phenotype that contributes to vascular remodeling and neointima formation in response to environmental cues such as vascular injury or inflammatory stimuli. Abnormal proliferation and migration of VSMCs is a hallmark of many occlusive vascular diseases, such as atherosclerosis, intimal hyperplasia and/or restenosis following percutaneous transluminal angioplasty (PTA), bypass surgery, and arterial-venous fistular (2–5). VSMCs exhibit differentiated and contractile phenotype in mature blood vessels, typically proliferating at an extremely low rate and having shallow synthetic activity (6). However, differentiated VSMCs can reversibly switch to a dedifferentiated state in response to mechanical insults, such as angioplasty, stenting, or bypass surgery (7). This phenotypic modulation is characterized by an increased rate of proliferation and migration, which contributes to intimal hyperplasia (8, 9). Therefore, finding therapies to effectively inhibit VSMCs proliferation, migration, and phenotype switching is a primary focus for preventing post-intervention complications in coronary and peripheral arterial diseases, along with endothelium protection and anti-thrombotic therapy.

It has been well documented that vascular injury induces the release of cytokines and growth factors to stimulate VSMCs proliferation and migration. The increased production of growth factors like PDGF-BB stimulates VSMCs proliferation, and migration (10) and modulates differentiated to dedifferentiated phenotype switching (11, 12) in response to vascular injury *via* initiating downstream signaling pathways. PDGF-BB, as a natural ligand of PDGFR-β (13), activates downstream signaling mitogen-activated protein kinases (MAPKs) and phosphatidylinositol 3-kinase (PI3K)/protein kinase B (AKT), which have been demonstrated to affect cell proliferation and migration (14–16). Moreover, previous studies have shown that PDGF-BB significantly inhibited the expression of VSMC differentiation markers, including α-SMA, SM22α, and calponin (17–19), through mechanisms including upregulation Kruppel-like factor 4 (KLF4). PDGF-PDGFR-MAPK-PI3K/AKT and KLF-myocardin pathways have attracted great interest as therapeutic targets for modulating VSMC pathophysiology and managing neointimal hyperplasia.

Pulsatilla chinensis (Bge.) Regel is a well-known and widely used herbal medicine in Asian countries attributed to its antimicrobial and anti-inflammatory properties (20, 21). Previous studies have shown that Pulsatilla chinensis saponins have a wide range of pharmacological effects, including anti-inflammatory, antioxidant, immunomodulatory, and cognitive enhancement (22). Saponins are the major ingredients in Pulsatilla. Among those, Anemoside B4 (B4) is one of the most abundant saponin compounds identified from the Pulsatilla chinensis (23). While most of the research has focused on the separation, extraction, pharmacokinetics, and pharmacodynamics of B4, a recent study reported that B4 could inhibit the phosphorylation of AKT and mTOR and induce apoptosis and autophagy of hepatocellular carcinoma (24). Another study has shown that Anemoside B4 prevents acute ulcerative colitis by inhibiting the TLR4/NF-κB/MAPK signaling pathway (25).

However, the role of B4 in modulating neointima hyperplasia and VSMC function is unknown. In the current study, we aimed to determine the effect of B4 on VSMC proliferation and migration and its ability to attenuate restenosis *in vivo*. Our data point to a model wherein B4 inhibits PDGF-BB-induced VSMC proliferation and migration, prevents the transformation of VSMCs from contractile to the synthetic phenotype, and attenuates neointima formation in a mouse acute femoral artery injury model through PI3K/Akt and p38 MAPK signaling. For the first time, our results reveal that B4 is a promising saponin to ameliorate occlusive vascular disease.

## Materials and Methods

### Reagents

Anemoside B4 (B4, chemical structure C_5_9H_9_6O_26_, molecular weight = 1,221.38, purity >98%) was purchased from the Chinese National Institute for the Control of Pharmaceutical and Biological Products. PDGF-BB was purchased from PEPROTECH. The small molecule inhibitors LY294002 (PI3K/Akt inhibitor), SD98059 (ERK inhibitor), SB203580 (p38 MAPK inhibitor), and SP600125 (JNK inhibitor) were obtained from Sigma-Aldrich (St. Louis, MO). Primary antibodies against Akt/phospho-Akt, ERK/phospho-ERK, p38 MAPK/ phospho-p38 MAPK, and JNK/phospho-JNK were from Cell Signaling Technology (Massachusetts, USA). Antibodies against α-SMA, Calponin, SM22-α, and BrdU were obtained from Abcam (Cambridge, UK). TUNEL staining kit was obtained from Roche. 5-Bromo-3′-deoxyuridine (BrdU) was purchased from Sigma-Aldrich (St. Louis, MO). See Supplementary Tables 1, 2 for detailed information.

### Animal and Mouse Femoral Artery Wire Injury

Six-week-old male C57BL/6J mice were purchased from the Jackson Laboratory. All experiments were performed in accordance with the guidelines/regulations and were approved by the Institutional Animal Care and Use Committee (IACUC) of Temple University. Mice were anesthetized with an intraperitoneal (IP) injection of ketamine (100 mg/kg) and xylazine (10 mg/kg). As previously described, the wire-induced left femoral artery injury was performed (26, 27). Mice were injected with B4 (20 mg/kg or 40 mg/kg) or equal volum of vehicle control (saline) daily intraperitoneally (IP) for 3 weeks. Mice were injected with BrdU subcutaneously (25 mg/kg) daily for 3 days and IP (30 mg/kg) 12 h before sacrifice. Femoral arteries were collected at the experimental endpoint and cryopreserved for histological staining. Cryosections (5 μm) were obtained for H&E, EVG, and BrdU staining. Morphometric analyses were performed as previously described (26, 28). Ten cross-sections were stained with H&E or EVG and used for lumen, neointimal, medial, and vessel area quantification. Each section was collected 100 μm apart to cover the whole injured vessel. Values were then averaged for each animal, and the mean was calculated from 5 to 6 animals per treatment.

### Primary VSMC Isolation and Culture

Primary mouse VSMCs (MVSMCs) were separated from the thoracic arteries of 6-week-old male C57BL/6J mice using collagenase, as previously described (28). In brief, mouse vessels were minced with a sterile blade and digested in a digestion mix (175 U/ml collagenase II, 1.25 U/ml elastase in 2.5 ml HBSS) by shaking at 500 rpm at 37°C for 30 min. The cell suspension was centrifuged at 920 rpm for 5 min, washed once with DMEM supplemented with 20% FBS, penicillin G (100 units/ml), and streptomycin sulfate (100 μg/ml). Plated cells on desired dishes in the same media. Cells were sub-cultured 1: 3 once confluent. Cells in passages 4–9 were used for all the experiments. The cells were starved with starvation media (0.3% FBS) for 24 h before experimental treatments.

### Histological Staining and BrdU Incorporation

Mice were anesthetized with an intraperitoneal injection of ketamine (100 mg/kg) and xylazine (10 mg/kg), then systemically perfused with PBS *via* the left ventricle. Isolated femoral arteries were fixed with 2%PFA in PBS for 24 h at 4°C, then kept in 30% sucrose in PBS until the next day at 4°C. Finally, tissues were embedded in OCT and kept at −80°C. Serial 5 μm sections were cut using a cryostat, and several morphometric and histological analyses were performed as previously described (29). H&E and EVG stained images were captured using inverted Olympus IX71microscopes and a digital camera. A BrdU incorporation assay measured the frozen mouse femoral arteries (3). Artery sections were washed with PBS, fixed with 2% PFA for 10 min, and incubated with 0.1% Triton-X100 for 20 min to permeabilize the cell membrane. Samples were neutralized by incubation in phosphate/citric acid buffer (pH 7.4) for 10 min at room temperature. Samples were then blocked with blocking buffer (5% normal goat serum, 0.5% BSA, 0.1% Triton-X100 in PBS) for 1 h. A primary anti-BrdU antibody was used at a 1:40 dilution. A FITC-labeled secondary antibody (anti-rat, 1:250) was used. Nuclei were stained with DAPI.

### Cell Proliferation and Viability Assay

Mouse VSMCs proliferation was examined by MTT assay and cell counting. MTT assays were performed as described previously with minor modifications (30). Briefly, MVSMCs (7,000 cells/well) were cultured overnight in a 96-well plate in starvation media for 24 h and pretreated with varying concentrations of Anemoside B4. Cells were then stimulated with PDGF-BB (10 ng/ml) in the absence or presence of different concentrations of Anemoside B4 or/and LY294002, SB203580. MTT stock solution (20 μl, 5 mg/ml in PBS) was added to wells at various time points, incubated for 4 h at 37 °C, and 200 μl DMSO was added to the media to solubilize the crystal for 10 min. The absorbance of cells was measured at 570 nm using a microplate reader. MVSMCs incubated with the starvation medium were used as controls. To obtain direct cell counts, MVSMCs (3 × 10^4^ cells/well) were cultured in 24-well and treated as described above, except for MTT addition. Cells were trypsinized and counted using a hemocytometer.

### TUNEL Staining for Detection of Cell Apoptosis

Mouse VSMCs were cultured on the pre-coated coverslips overnight and treated with the conditions described in the cell proliferation assay. TUNEL and Fluor 488 Apoptosis detection assay (Roche) was performed following the manufacturer's protocol to detect VSMC apoptosis. DAPI was used as the nuclear DNA marker.

### Wound Healing Assays

Mouse VSMCs were seeded into 12-well plates, cultured up to 85% confluency, and then starved (0.3% FBS) for 24 h. Cell monolayers were scratched using a 1 ml pipette tip (31). Cells were then treated with PDGF-BB (10 ng/ml) in the absence or presence of different concentrations of Anemoside B4, LY294002, SD98059, SB203580, SP600125, or combination for 12 or 24 h. Images were captured at 0, 12, and 24 h with inverted Olympus IX71microscopes and a digital camera. Quantification was made by using ImageJ.

### Modified Boyden Chamber Assay

Three-dimensional cell migration was performed using the 8 μm pore size polycarbonate filters modified Boyden chamber (Costar, 3422) transwell assay. MVSMCs were plated at a density of 3 × 10^4^ cells/well cultured in starvation media in the upper chamber pre-coated with 0.1% gelatin. PDGF-BB (10 ng/ml) and varying concentrations. Anemoside B4 was added to the lower chamber. Cells in starvation media or PDGF-BB served as controls. Twelve hours later, the migrated cells in the lower chamber were fixed with 2% PFA for 20 min and then stained with 0.1% crystal violet for another 20 min. The five views migrated cells from each well were captured and quantified using ImageJ (NIH).

### Western Blotting

Cells were briefly washed with cold PBS and scraped in cold 1 × TBSN buffer supplemented with protease inhibitor and phosphatase inhibitors on ice. Cell lysates were centrifuged at 12,000 × g for 30 min at 4°C. Supernatants were mixed with loading buffer and denatured at 95°C for 5 min. Equal amounts of protein from each sample were separated on 10%SDS-PAGE gels, transferred onto nitrocellulose membranes and block with 5%BSA 1 h, and then immunoblotted with primary antibodies at 4°C overnight and secondary antibodies (IRDye) at Room temperature for 1 h. Digital images were taken with a Gel DocTM XR+ System using Image Lab software (BioRad). Densitometry of western blot bands was quantitated using ImageJ.

### RNA Extraction and Quantitative Real-Time (RT)-PCR Analysis

According to the manufacturer's protocol, the MVSMCs were briefly washed with cold PBS, and total RNA was extracted using Trizol (Invitrogen, Camarillo, USA). One μg of total RNA was reverse transcribed to cDNA using the iScript reverse transcript synthesis kit (Bio-Rad). cDNA was then used for quantitative real-time RT-PCR analysis (Bio-Rad CFX96 Real-Time System) using SYBR Green supermix (Bio-Rad). The mRNA level was normalized to ribosomal RNA 18S as a housekeeping gene. Real-time RT-PCR was analyzed using the 2^−ΔΔCT^ method (CT, comparative threshold cycle). CT values were normalized to the internal control 18s for MVSMCs samples. ΔΔCT = (CT experimental gene – CT housekeeping gene) – (CT control gene-housekeeping gene). All samples were run in triplicates.

### Statistical Analysis

Values are presented as mean ± SEM from at least three independent Experiments. GraphPad Prism Software version 8.0 was used for statistical analysis. One-way ANOVA followed by Tamhane T2 test to correct for multiple comparisons was used to perform multiple groups comparisons. Two-tailed Student's-test was used to analyze the differences between the two groups. *P* < 0.05 were considered statistically significant.

## Results

### Animoside B4 Attenuates Neointima Formation Induced by Femoral Artery Injury *in vivo*

**Figure 1:**
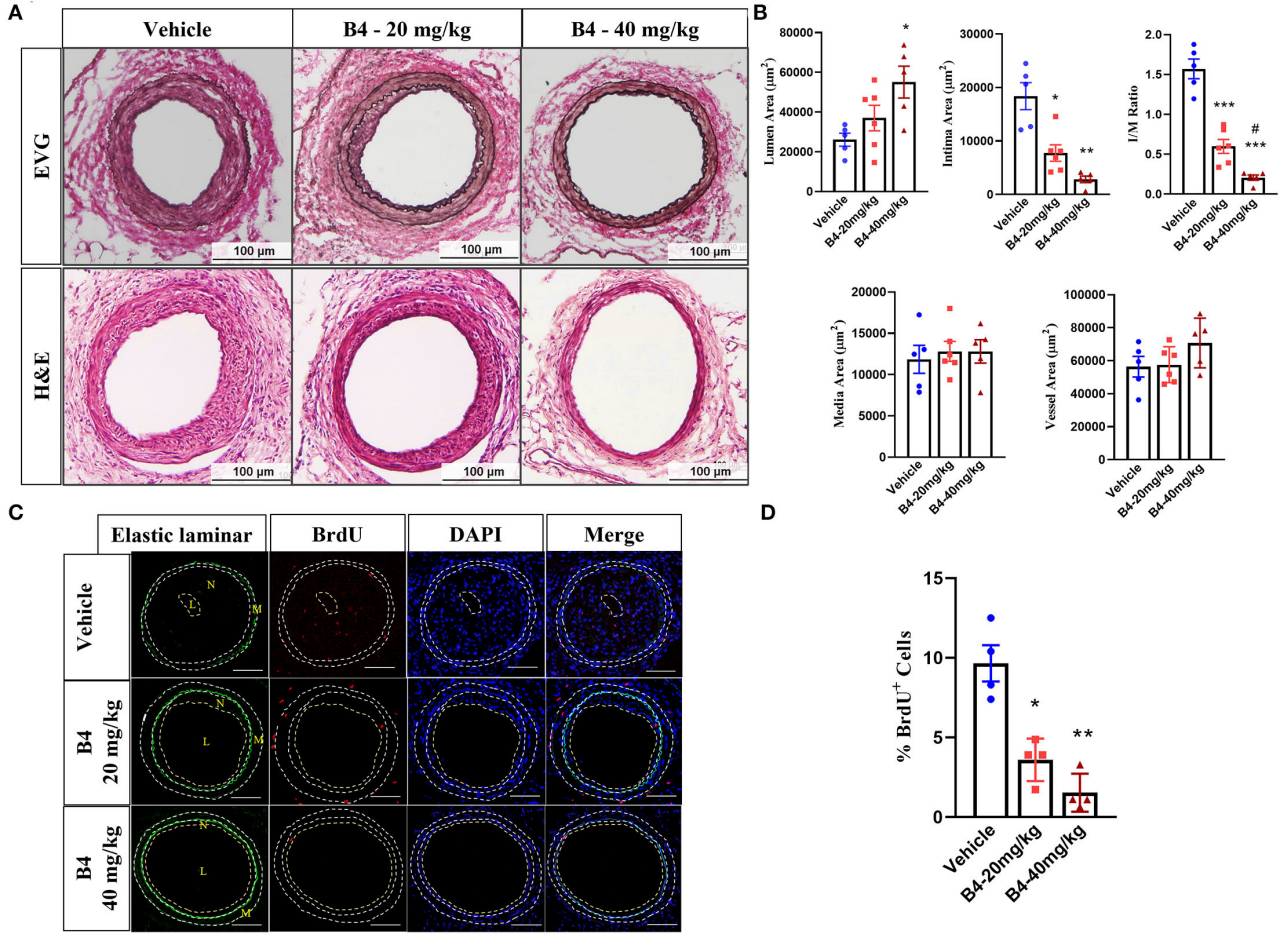
B4 attenuates neointima formation in a murine femoral artery wire injury model. Six-week-old C57BL/6J male mice were intraperitoneally injected with B4 (20 mg/kg/d), (40 mg/kg/d), or saline (Vehicle) daily for 3 weeks. **(A)** Elastic van Gieson (EVG, upper panel) and H&E staining (lower panel) staining of injured femoral arteries (Vehicle) and B4 (20 mg/kg/d), (40 mg/kg/d). Scale bar, 100 μm. **(B)** Morphometric analysis of lumen area, neointima area, intima-to-media ratio, media area, and vessel area of injured femoral arteries from mice injected with vehicle or B4. **(C)** Representative BrdU immunofluorescence stained images of the sections from vehicle or B4. The dotted line represents the auto-fluorescent of elastic laminar. Scale bar, 100 μm. **(D)** The percentage of BrdU positive cell number was quantified in sections from sham-operated and injured femoral arteries from vehicle or B4 treated mice. Cells in the media and neointima layers (between the external elastic laminar and the lumen of each section) were included. Data shown are means ± SEM. *N* = 5 for control and *N* = 6 for B4 treatment. *P < 0.05; **P < 0.01; ***P < 0.001; compared with Vehicle group. #*P* < 0.05; compared with B4-20mg/kg group.

To determine whether B4 (chemical structure shown in Supplementary Figure 1) affects neointima formation, we performed the mouse femoral artery wire injury model in C57BL/6J mice treated with vehicle or B4. In this model, the endothelial layer of the femoral artery is denuded by passaging an angioplasty guided wire three times, and extensive neointima is formed as a result of medial VSMC proliferation and migration (26, 27). No difference in the gross vessel morphology was observed in femoral arteries of mice given a systemic injection of vehicle or B4 (not shown). A robust neointima formation was observed in vehicle-treated mice 3 weeks following femoral arterial wire injury. The treatment of B4 exhibited a significant reduction in intimal thickness and intima to media ratio compared to the vehicle treatment (Figures 1A,B). The media area and vessel area determined by internal and external elastic laminar were not changed between groups (Figures 1A,B). Following injury, pathological vascular remodeling is attributed to increased VSMC proliferation (1, 32). We evaluated VSMC proliferation by BrdU incorporation *in vivo*. As shown in Figure 1C, we observed growing numbers of BrdU labeled VSMC in the injured vehicle-treated femoral arteries (Figure 1C, top panel). Notably, B4 treatment significantly reduced the number of BrdU-positive cells detected in the media and neointima layers of the injured vessels (Figure 1C, middle and bottom panels, and Figure 1D). Together, these results indicate that B4 treatment significantly reduced neointima formation by inhibiting VSMC proliferation *in vivo*.

### B4 Inhibits PDGF-BB Induced VSMC Proliferation and Migration but Does Not Affect Cell Viability and Apoptosis

**Figure 2:**
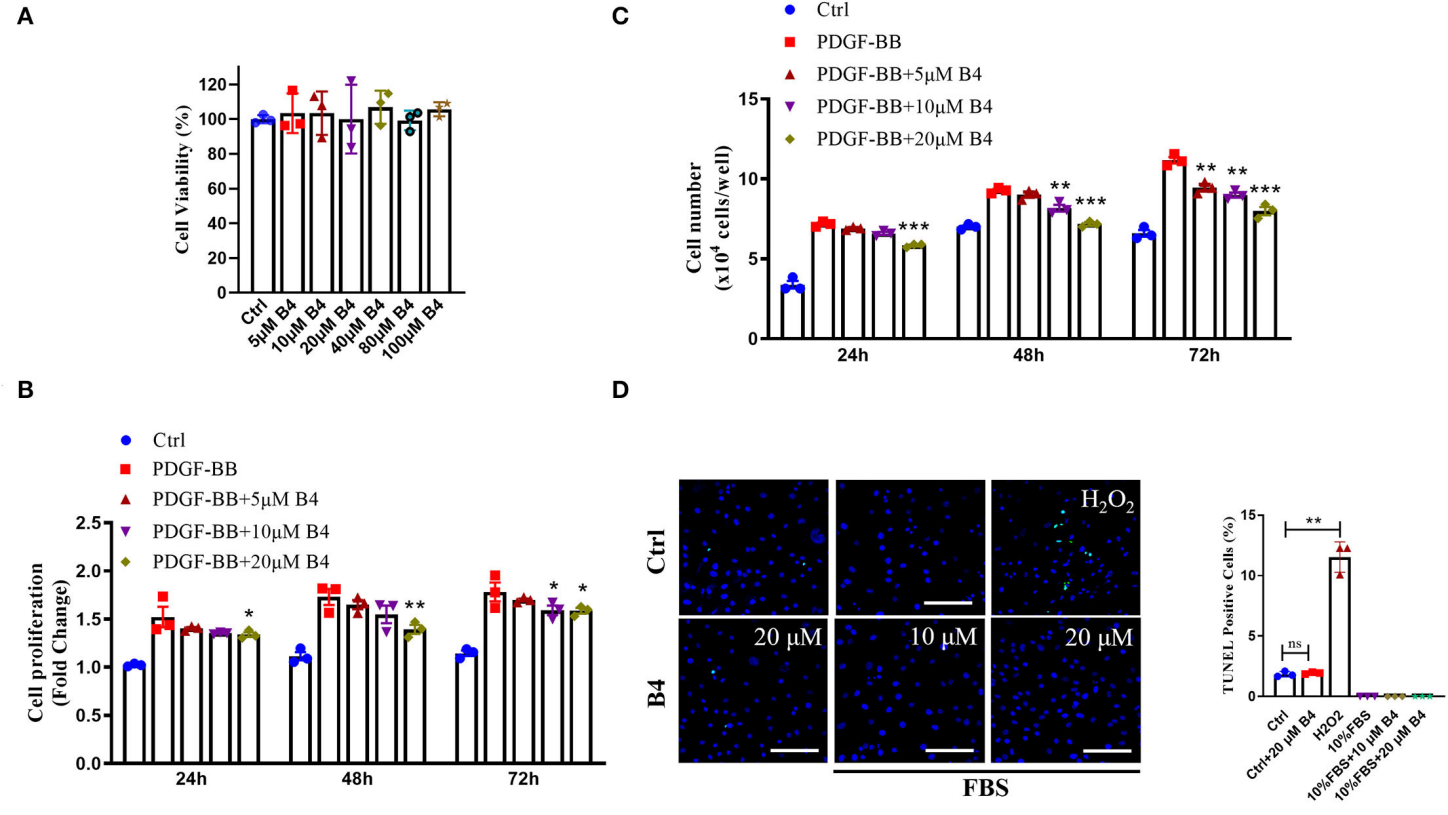
B4 inhibits MVSMCs proliferation but not cell viability or apoptosis. **(A)** Cell viability was measured by MTT assays. PDGF-BB-deprived MVSMCs were pretreated with 5–100 μM of B4 for 24 h and incubated with/without PDGF-BB in the absence or presence of B4 for the indicated times. Cell proliferation was detected by **(B)** MTT assays. **(C)** Direct cell counting. **(D)** MVSMC apoptosis was measured by TUNEL staining. Data shown are means ± SEM. *N* = 3. *P < 0.05; **P! < 0.01; ***P < 0.001; compared with PDGF-BB control group. Scale bar, 100 μm. All experiments were repeated at least for three times.

To examine the role of B4 on VSMC function *in vitro*, we first determined its toxicity. MTT assay showed that B4 did not affect MVSMC viability even at the highest concentration (100 μM) tested at 24, 48 h (Supplementary Figure 2), and 72 h (Figure 2A) after treatment. The abnormal proliferation and migration of VSMCs play a pivotal role in developing neointimal hyperplasia after vascular injury (27, 29). To investigate the possible mechanisms underlying B4's ability to attenuate neointima hyperplasia, MVSMCs were stimulated with or without PDGF-BB in varying concentrations (0, 5, 10, and 20 μM) of B4, and cell proliferation, migration, and apoptosis assays were performed. MVSMCs cultured without PDGF-BB proliferated at a standard rate, and B4 did not affect MVSMC proliferation under these basal conditions. Proliferation was significantly increased with the addition of PDGF-BB, and this response to PDGF-BB was attenuated by B4 treatment in a time- and dose-dependent manner (Figures 2B,C). To exclude the possibility that the anti-proliferation effect of B4 on MVSMC was due to increased apoptosis, we examined MVSMC apoptosis under both basal (0.3% FBS) and stimulated (20% FBS) conditions. TUNEL staining showed B4 did not increase MVSMCs apoptosis (Figure 2D). These results suggest that B4 inhibits PDGF-BB-induced VSMC proliferation but does not affect cell cytotoxicity or apoptosis.

**Figure 3:**
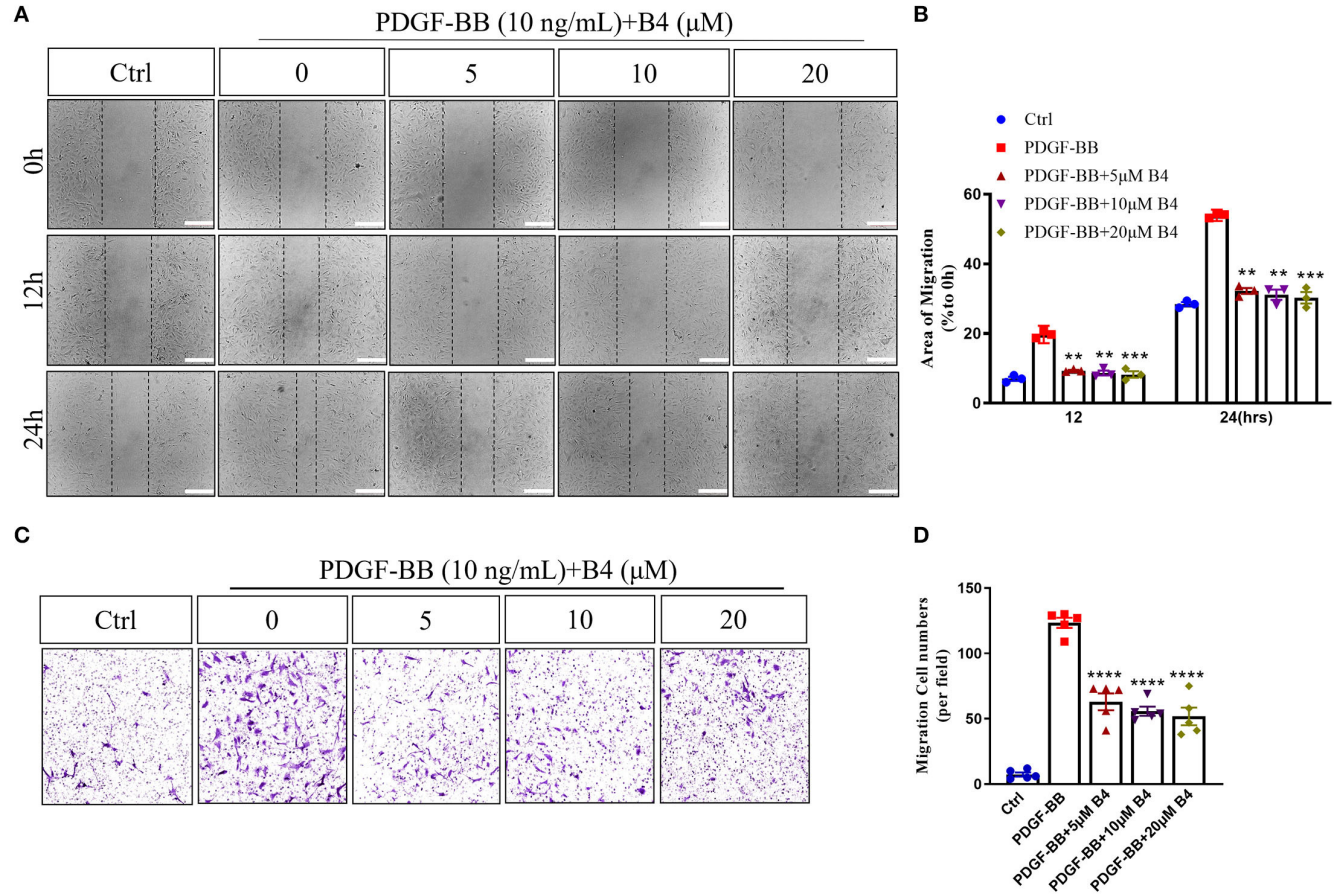
B4 prevents MVSMCs migration. **(A)** Representative images of quiescent MVSMCs were pretreated with B4 for 24 h and stimulated with PDGF-BB for 24 h. Images were captured at 0, 12, 24 h. The percentage of area covered by migrated cells over time was calculated using ImageJ **(B)**. *N* = 3. Scale bar, 200 μm. **(C)** Representative images from transwell migration assay with different conditions. **(D)** Quantification for the number of migrated cells is shown in the bar graph. *N* = 5. All experiment was repeated at least three times. Data shown are means ± SEM. **P < 0.01; ***P < 0.001; ****P < 0.0001 vs. PDGF-BB control group.

In addition to proliferation, VSMC migration is another critical process in promoting neointimal hyperplasia (33). PDFG-BB is an effective growth factor that could increase VSMC migration (10). To determine the potential effect of B4 on MVSMC migration, we performed 2D wound healing and 3D transwell migration assays. Figures 3A,B shows that B4 dose-dependently inhibited PDGF-BB induced MVSMC migration and wound closure. The transwell assay further indicated the inhibitory effect where B4 dose-dependently inhibited the PDGF-BB-induced MVSMC transmigration (Figures 3C,D). These results suggest that B4 inhibits VSMC proliferation and migration but does not adversely affect cellular viability.

### B4 Abrogates PDGF-BB-Induced VSMC Dedifferentiation

**Figure 4:**
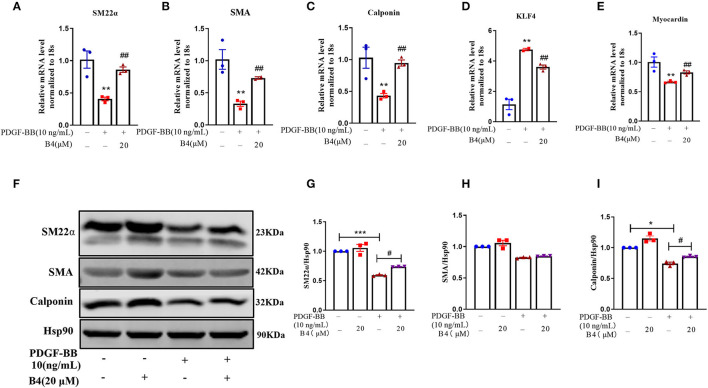
B4 abrogates PDGF-BB-induced MVSMCs dedifferentiation. MVSMCs were pretreated with B4 (20 μM) for 24 h, followed by PDGF-BB (10 ng/ml) stimulation for 48 h. **(A–E)** Bar graph showing the relative mRNA level of SM22α, SMA, Calponin, KLF-4, and Myocardin. **(F)** Western blot was employed to quantitate the expression levels of contractile protein SM22α, SMA, Calponin. Hsp90 was used as a loading control. **(G–I)** Bar graph showing the relative protein level of SM22α, SMA, and Calponin. Values are mean ± SEM. *P < 0.05; **P < 0.01; ***P < 0.001 vs. Control, ^#^*P* < 0.05; ^##^*P* < 0.01 vs. PDGF-BB. *N* = 3 for each group.

Vascular smooth muscle cells in mature, healthy blood vessels are highly specialized cells with a quiescent, differentiated, and contractile phenotype. They express high levels of contractile proteins such as SMA, SM22α, and calponin. In response to vascular injury, VSMCs switch to a dedifferentiated, proliferative, and migratory phenotype (synthetic phenotype), with decreased levels of contractile proteins (34, 35). To test whether B4 affects PDGF-BB-induced VSMC dedifferentiation, after starvation, MVSMCs were stimulated with PDGF-BB (10 ng/ml) and 20 μM B4, alone or in combination for 48 h. The results showed that PDGF-BB promoted the MVSMC phenotypic alteration from a differentiated to a dedifferentiated status, as evidenced by decreased VSMC contractile genes SMA, calponin, and SM22α at mRNA (Figures 4A–C) and protein levels (Figures 4F–I). B4 treatment reversed PDGF-induced MVSMC contractile gene down-regulation. KLF4 and myocardin are master regulators of VSMC plasticity (36–38). We examined whether B4 regulates VSMC contractile genes through KLF4 and myocardin. As shown in Figures 4D,E, B4 reversed the effect of PDGF-BB in the upregulation of KLF4 and downregulation of myocardin. Our study suggests that B4 antagonizes VSMC dedifferentiation through the transcriptional regulation of KLF4 and myocardin.

### B4 Inhibits PDGF-BB-Induced VSMC Proliferation and Migration Through p38 MAPK and PI3K/Akt Signaling Pathways

**Figure 5:**
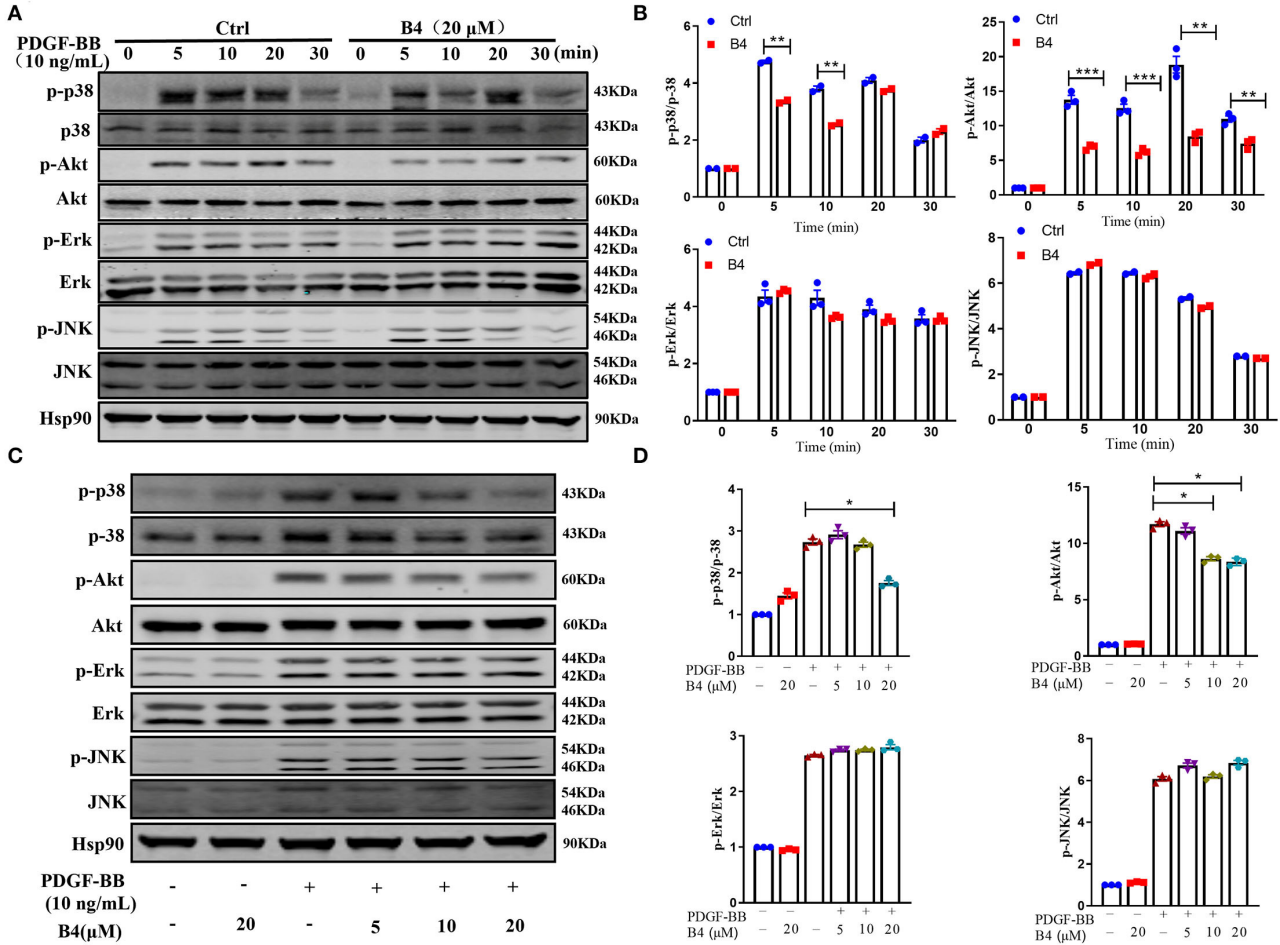
B4 inhibits PDGF-BB-induced PI3K/Akt and p-38 MAPK signaling pathway. **(A)** Western blot analysis of p-Akt/Akt, p-p38/p38, p-ERK/ERK, p-JNK/JNK in MVSMCs treated with B4 for 24 h and stimulated with PDGF-BB for 0–30 min. Hsp90 was used as the loading control. **(B)** Densitometry analysis of the phosphorylation protein normalized to the total protein levels. **(C)** Western blot analysis of MVSMCs treated with different concentrations of B4 (0, 5, 10, 20 μM) for 24 h and stimulated with PDGF-BB 10 ng/ml for 15 min. The protein expression levels of p-p38/p38, p-Akt/Akt, p-ERK/ERK, and p-JNK/JNK were blotted. Hsp90 was used as a loading control. Representative western blots from three experiments are shown. **(D)** Densitometrical analysis of the western blots. Data shown are means ± SEM. *N* = 3. *P < 0.05; **P < 0.01; ***P < 0.001; compared to non-stimulated control group. All experiments were repeated at least for three times.

It is well known that phosphorylation and subsequent activation of MAPK and PI3K/Akt are the major signals involved in PDGF-BB stimulated VSMC proliferation and migration (39, 40). To determine the effect of B4 on these signaling pathways, the phosphorylation of Akt and MAPKs was examined in MVSMCs stimulated with PDGF-BB. We observed a rapid decrease in p38 MAPK and Akt phosphorylation, but not ERK1/2 or JNK activation, in MVSMCs following B4 treatment in a time- and dose-dependent manner (Figure 5). The total protein levels of Akt and MAPKs were unaffected by PDGF-BB or B4 treatment.

**Figure 6:**
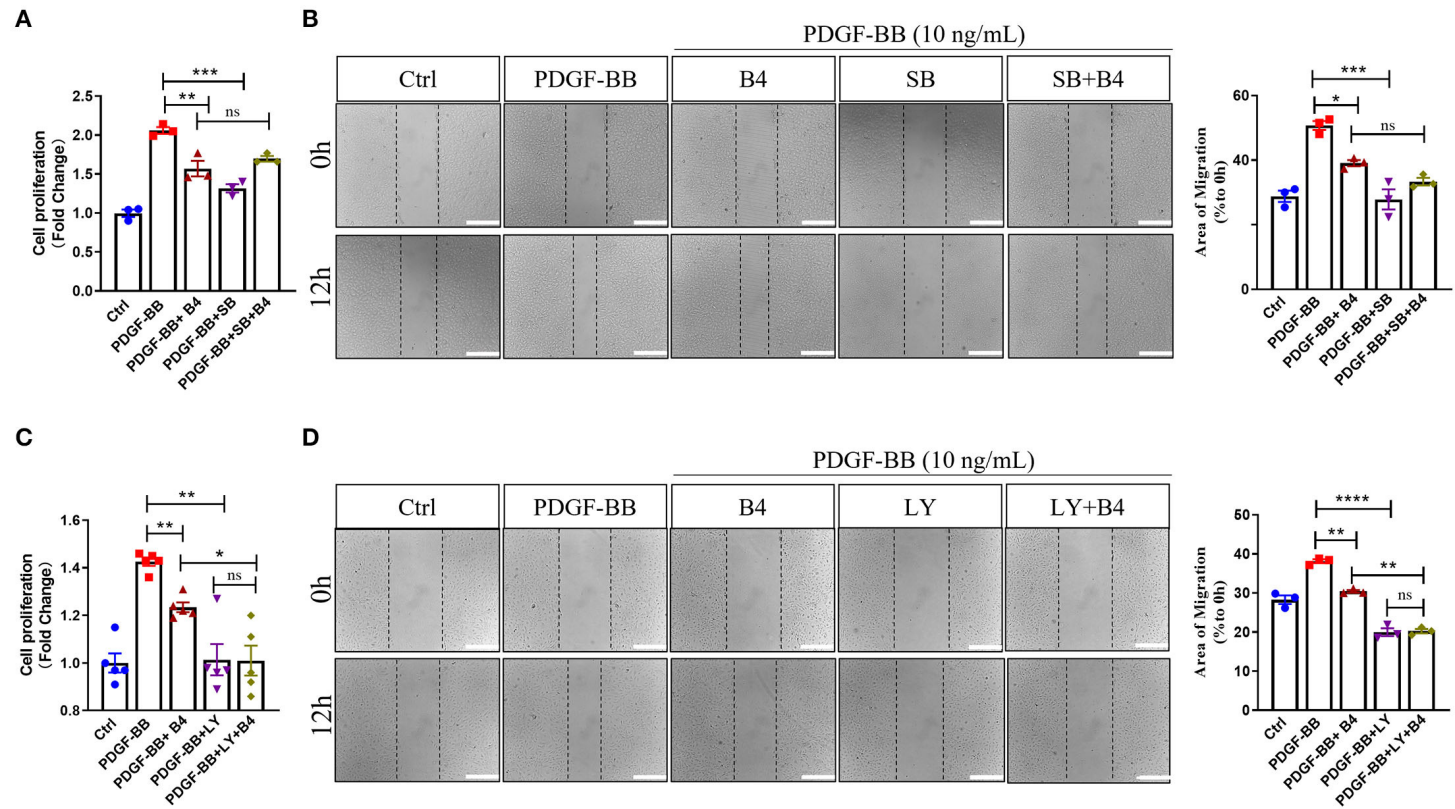
B4 inhibits cell proliferation and migration by targeting the p38 MAPK and PI3K/Akt signaling pathways in VSMC. Quiescent MVSMCs were treated with B4 (20 μM) for 24 h and incubated with SB203580 (20 μM) or SB203580 and B4 in the presence or absence of PDGF-BB for 48 h. **(A)** Cell proliferation was quantified as a fold increase of control. **(B)** Cell migration was quantified as the percentage of the area of migrated cells covered area relative to time 0. Quiescent MVSMCs were treated with B4 (20 μM) for 24 h and incubated with LY294002 (10 μM) or LY294002 and B4 in the presence or absence of PDGF-BB for 48 h. **(C)** Cell proliferation was quantified as a fold increase of control. **(D)** Cell migration was quantified as the percentage of the area of migrated cells covered area relative to time 0. Data shown are means ± SEM. *P < 0.05; **P < 0.01; ***P < 0.001 compared with PDGF-BB control group. All experiments were repeated for at least three times.

To further determine that B4 inhibits MVSMC proliferation and migration through p38 MAPK and PI3K/Akt signaling, we tested the effect of inhibiting p38 MAPK, PI3K/Akt, ERK, or JNK signaling in cultured MVSMC with their respective specific small molecule inhibitors SB203580, LY294002, PD98059, and SP600125. We compared VSMC proliferation, migration, MAPK, and Akt activity in MVSMC at baseline, with PDGF-BB stimulation in the presence of B4, treated with the pharmacological inhibitors, or both. MVSMC proliferation and migration were significantly reduced in the presence of B4 or p38 MAPK inhibitor SB203580 compared to PDGF-BB treated cells. Treatment of B4 with SB203580 showed no synergistic or additive effect on VSMC proliferation and migration (Figures 6A,B). Similarly, MVSMC proliferation and migration were significantly attenuated in the presence of B4 or Akt inhibitor LY294002 compared to PDGF-BB treated cells. Notably, co-treatment of MVSMC with LY294002 and B4 showed that B4 had no effect in further reducing cell proliferation compared to LY294002 alone (Figure 6C). The same observations were found for migration and Akt activity (Figure 6D and Supplementary Figures 3A,B). However, co-treatment of B4 with the ERK or JNK inhibitors showed a synergistic effect on VSMC proliferation and/or migration (Supplementary Figures 3C–G). These data suggest that B4 inhibits VSMC proliferation and migration, at least in part, through p38 MAPK and PI3K/Akt but not ERK or JNK signaling pathways.

Our results indicate that B4 targets the p38 MAPK and PI3K/Akt signaling pathway and inhibits cell proliferation and migration in VSMC, contributing to the attenuation of neointima hyperplasia.

## Discussion

In the present study, we used a preclinical mouse model of endothelial denudation-induced vascular neointima hyperplasia to examine whether a novel saponin, Anemoside B4, has potential pharmacological effects on preventing neointima formation and vascular remodeling. B4 significantly attenuated neointima formation by inhibiting VSMC proliferation *in vivo*. We demonstrated that B4 inhibits VSMC proliferation and migration by inhibiting the p38 MAPK and PI3K/Akt signaling pathways and regulating the VSMC contractile gene expression. These results provided the molecular basis by which B4 exerted a protective role in VSMC biology.

It is well known that dysregulated VSMCs play a critical role in vascular restenosis, atherosclerosis and hypertension (41). Unlike skeletal or cardiac muscle cells, VSMCs are not terminally differentiated, and their phenotype can be modulated between contractile and synthetic states in response to environmental stimuli (42). The phenotypic switching of VSMCs is one of the major cellular events for the proliferation and migration of VSMCs. Increased VSMC proliferation and migration are essential to atherosclerosis, postangioplasty restenosis, and hypertension (43). Therefore, restenosis treatments after PTA have mainly focused on inhibiting VSMC proliferation and migration. Drugs such as Rapamycin (sirolimus) and Paclitaxel (Taxol), which inhibit VSMC proliferation and migration, have been widely used in drug-eluting stents to prevent in-stent restenosis (44, 45). Our current study indicated that B4 attenuated neointimal hyperplasia induced by endothelium denudation injury while maintaining the lumen diameter (Figures 1A,B). The therapeutic benefit of B4 lies in its ability to inhibit excessive VSMC proliferation, as demonstrated by decreased BrdU incorporation (Figures 1C,D, 2C,D).

The healthy and mature VSMCs express high-level contractile proteins, including α-SMA, SM22α, and calponin, which significantly regulate vascular tone and blood pressure (43). VSMCs have remarkable plasticity without terminally differentiated properties (34). In response to the environmental stimuli, VSMCs undergo phenotypic switching from a differentiated and contractile state to a synthetic state (2). The phenotypic switch of VSMC is one of the major cellular events underlying many VSMC-related pathological conditions like restenosis (43). In the current study, we observed that B4 pretreatment prevented PDGF-BB induced VSMC differentiation, as reflected by increased VSMC contractile genes α-SMA, Calponin, and SM22α at both mRNA (Figures 4A–C) and protein levels (Figures 4F–I). KLF4 is a potent repressor of differentiation markers involving binding to the TGF-β control element within the promoter/enhancer regions of VSMC differentiation genes. KLF4 also interacts with serum response factor (SRF) and represses the expression of an SRF coactivator and VSMC differentiation master regulator, myocardin (36, 46, 47). B4 reduced KLF4 and elevated myocardin expression in mRNA levels (Figures 4D,E). These findings may represent a mechanism by which B4 inhibited VSMC dedifferentiation through the regulation of KLF4. However, further study is required to reveal its upstream mediators.

An abnormal increase in VSMC proliferation and migration is pivotal to neointima development in post-angioplasty restenosis. Inhibiting the VSMC migration and proliferation may be a plausible strategy for controlling the neointima hyperplasia in vascular remodeling-related disorders (48, 49). Our results indicate that the B4 inhibitory affects PDGF-BB-induced VSMC proliferation and migration (Figures 3A–C), protecting against neointimal hyperplasia *in vivo* (Figure 1). Our study provides the first direct evidence for the inhibitory effect of the B4 on PDGF-BB-induced VSMC migration, proliferation, and pathological vascular remodeling processes such as neointimal hyperplasia.

Numerous studies have indicated that the MAPK pathway, including p38 MAPK, ERK1/2, and JNK pathways, plays a crucial role in VSMC differentiation and proliferation (50). Inhibition of p38 MAPK decreased PDGF–BB–induced VSMC proliferation and neointimal formation after vascular injury (51). In the present study, we found that B4 blocked p38 MAPK activation induced by the stimulation of PDGF-BB (Figure 5). The p38 MAPK inhibition or B4 inhibited VSMC proliferation and migration. SB203580 had no additive effect when used with B4 treatment (Figures 6A,B), indicating B4 inhibits VSMC proliferation and migration, at least in part, through p38 MAPK inhibition.

PI3K/Akt pathway also plays a pivotal role in growth factors or cytokines (such as PDGF, interleukin-1, and TNF-α) induced VSMC proliferation and migration (15, 16, 40). The current study shows that B4 attenuated PDGF-BB-induced Akt phosphorylation but not ERK1/2, JNK activation (Figure 5). Furthermore, pharmacological inhibition of PI3K/Akt, but not antagonizing ERK or JNK, abolished the effect of B4 on VSMC proliferation and migration (Figures 6C,D and Supplementary Figures 3C–G). These results suggest B4 modulates VSMC function primarily through Akt signaling. Akt kinase is essential in many cellular processes, including proliferation, migration, cell growth, and metabolism. There are three known Akt isoforms, Akt1, Akt2, and Akt3, which play critical and diverse roles in the cardiovascular system (52). Akt1 is the major isoform expressed in VSMCs, endothelial cells, and macrophages, mediating cell survival and proliferation (53). Akt2 is required for rapamycin-induced VSMC differentiation (54), while Akt3 is mainly localized to the brain and testes (55, 56). The current study demonstrated that B4 inhibits VSMC proliferation and migration by inhibiting Akt activation. However, further studies are needed to determine whether the anti-proliferation and anti-migration effect of B4 is Akt isoform-specific.

**Figure 7:**
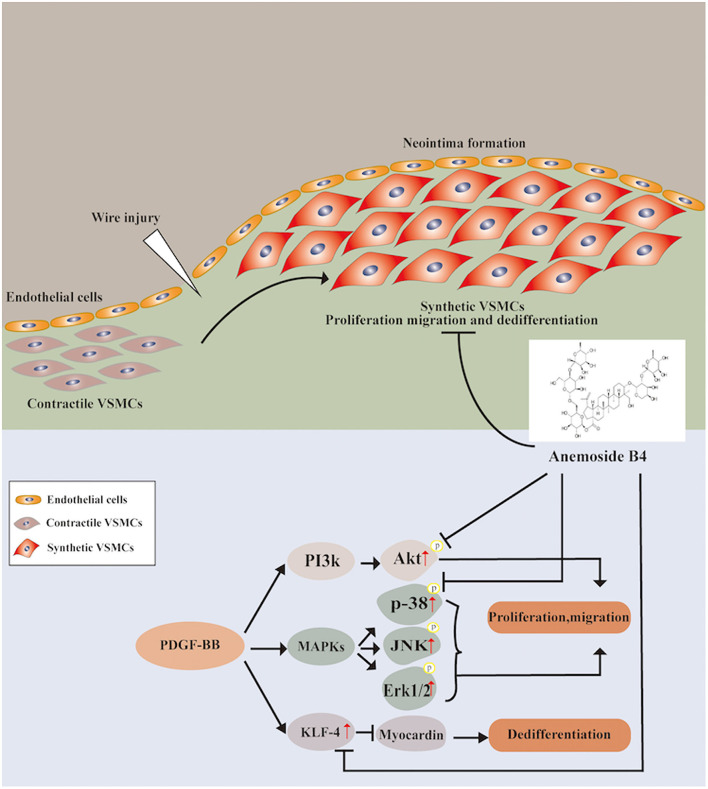
Schematic diagram of the potential mechanisms of B4 in regulating PDGF-BB-induced VSMC proliferation, migration, and dedifferentiation.

In conclusion, for the first time, the present study provides evidence that B4 attenuates neointimal hyperplasia in response to arterial injury *in vivo*. B4 inhibits growth factor-induced VSMC proliferation, migration, and dedifferentiation. The dual regulatory effect on the p38 MAPK and PI3K/Akt pathways provides the first insight into the molecular mechanisms underlying the vascular remodeling effect of Anemoside B4 (Figure 7). Our findings shed light on the clinical use of B4 in managing vascular restenosis.

## Data Availability Statement

The original contributions presented in the study are included in the article/Supplementary Material, further inquiries can be directed to the corresponding author.

## Ethics Statement

The animal study was reviewed and approved by IACUC Committee, Temple Univesity.

## Author Contributions

JY designed the study and coordinated all experimental work. DS, PQ, CZ, LH, GZ, YF, and JY carried out the experimental work. DS, PQ, QZ, GZ, WH, YF, SY, X-fY, and JY analyzed and interpreted the data. DS and JY wrote the manuscript with valuable input from all other authors. All authors have approved the submitted version of the manuscript.

## Funding

This work was supported by an award from the American Heart Association (18EIA33900065) to JY. DS and CZ were supported by Jiangxi Key Laboratory of Traditional Chinese Medicine for Prevention and Treatment of Vascular Remodeling Related Diseases (No. 20202BCD42014).

## Conflict of Interest

The authors declare that the research was conducted in the absence of any commercial or financial relationships that could be construed as a potential conflict of interest.

## Publisher's Note

All claims expressed in this article are solely those of the authors and do not necessarily represent those of their affiliated organizations, or those of the publisher, the editors and the reviewers. Any product that may be evaluated in this article, or claim that may be made by its manufacturer, is not guaranteed or endorsed by the publisher.
