# Supplementary material for: Anemoside B4 Inhibits Vascular Smooth Muscle Cell Proliferation, Migration, and Neointimal Hyperplasia
Source: Front Cardiovasc Med. 2022 May 10;9:907490. doi: 10.3389/fcvm.2022.907490 (PMC9127303; doi:10.3389/fcvm.2022.907490)
Supplement: Supplementary file 1 [file Data_Sheet_1.PDF]

Anemoside B4 inhibits vascular smooth muscle cell proliferation, migration, and neointimal hyperplasia

Dan Shan[1,2,3], Ping Qu[2], Chao Zhong[1,2], Luling He[1], Qingshan Zhang[3], Guoyue Zhong[4], Wenhui Hu[2], Yulin Feng[5], Shilin Yang[5], Xiao-feng Yang[2], Jun Yu[2,*]

[1]Center for Translational Medicine, Jiangxi University of Traditional Chinese Medicine, Nanchang, Jiangxi 330002, China;

[2]Department of Cardiovascular Sciences and Center for Metabolic Disease Research, Lewis Katz School of Medicine, Temple University, Philadelphia, PA 19140, USA;

[3]Affiliated Hospital of Inner Mongolia University for the Nationalities, Tongliao, Inner Mongolia 028000, China;

[4]Research Center of Natural Resources of Chinese Medicinal Materials and Ethnic Medicine, Jiangxi University of Traditional Chinese Medicine, Nanchang, Jiangxi 330002, China;

[5]The National Pharmaceutical Engineering Center (NPEC) for Solid Preparation in Chinese Herbal Medicine, Nanchang, Jiangxi 330006, China.

[*]Correspondence

Jun Yu, MD
Department of Cardiovascular Sciences and Center for Metabolic Disease Research,
Lewis Katz School of Medicine, Temple University
Philadelphia, PA 19140, USA
Tel: +1-215-707-3286
Email: jun.yu@temple.edu

Supplementary Figure 1

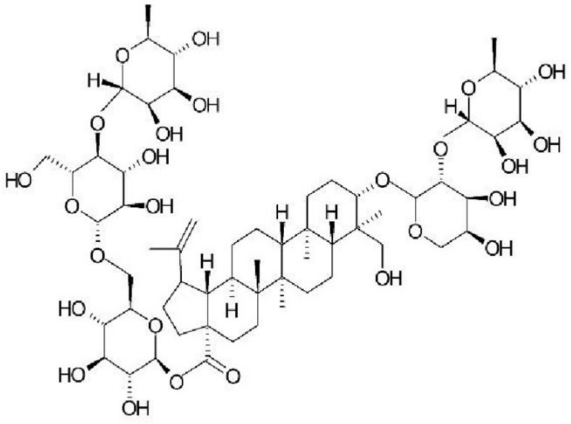

Supplementary Figure 1. The chemical structure of Anemoside B4.

Supplementary Figure 2

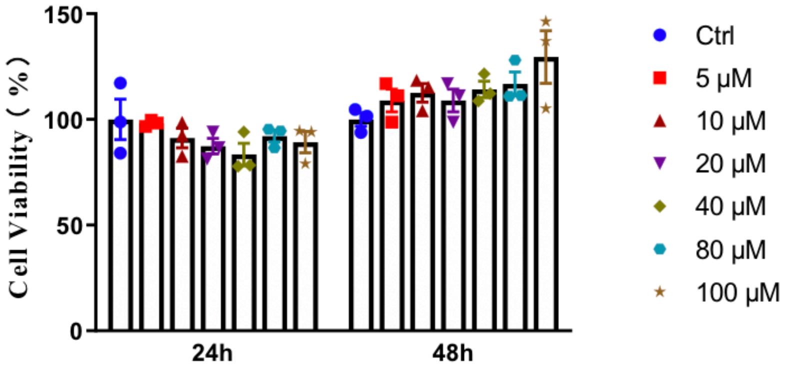

Supplementary Figure 2. Animoside B4 does not influence VSMC viability. MVSMCs were grown to reach confluency. Cell viability was measured by MTT assays at 24 and 48 hours. Data shown are means ± SEM. N = 3. *P < 0.05 compared with control group. All experiments were repeated at least for 3 times.

Supplementary Figure 3

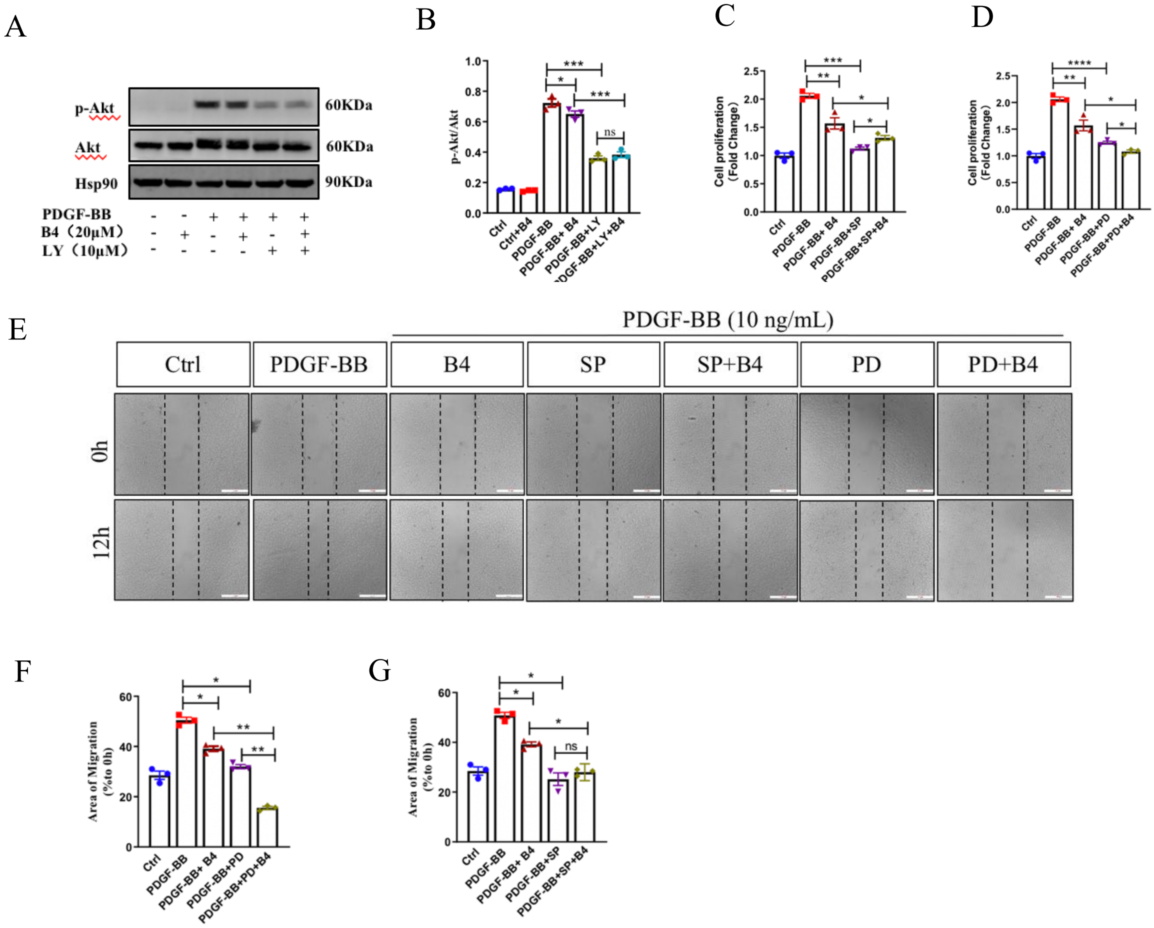

Supplementary Figure 3. B4 inhibits PDGF-BB-induced PI3K/Akt and p-38 MAPK, but not ERK or JNK signaling pathway. (**A**) Western blot of p-Akt and total Akt from MVSMCs treated with PDGF-BB, PDGF-BB + B4 with or without LY294002. (**B**) Bar graph showing the densitometry analysis of phosphorylated Akt protein normalized to the total Akt levels. N=3 for each condition. (**C-D**) Quiescent MVSMCs were treated with B4 (20 μM) for 24 hours and incubated with SP600125 (20 μM), PD98059 (50 μM), in combination of B4 (20 μM), or vehicle 48 hours. Cell proliferation was determined by MTT assay. (**E**) Cells migration was determined by scratch wound assay. (**F-G**) Quantification for the number of migrated cells. Data shown are means ± SEM. *P < 0.05; **P < 0.01; *** P < 0.001; **** P < 0.0001. All experiments were repeated at least for 3 times.

Supplementary Table 1: Antibody list

Antibody	Company	Catalog #	Species	Dilution	Application
Hsp90	BD	610419	Mouse	1:1000	Western
p-Akt(Ser473)	Cell Signaling	4051	Mouse	1:1000	Western
Akt	Cell Signaling	4685	Rabbit	1:1000	Western
p-Erk	Cell Signaling	4377	Rabbit	1:1000	Western
Erk	Cell Signaling	4696	Mouse	1:1000	Western
p-p38	Cell Signaling	9216	Mouse	1:500	Western
p38	Cell Signaling	9212	Rabbit	1:500	Western
p-JNK	Cell Signaling	4668	Rabbit	1:1000	Western
JNK	Santa Cruz	sc-7345	Mouse	1:500	Western
Calponin	Abcam	ab46794	Rabbit	1:2000	Western
SM22a	Abcam	ab14106	Rabbit	1:1000	Western
SMA	DAKO	M0851	Mouse	1:1000	Western
IRDYE 680RD	Odyssey	926-68073	Rabbit	1:5000	Western
IRDYE 800CW	Odyssey	926-32212	Mouse	1:5000	Western
Anti-BrdU	Abcam	ab6326	Rat	1:40	IF

Supplementary Table 2: Other reagent list

Reagents	Company	Cat log#
Anemoside B4	Chinese National Institute for the Control of Pharmaceutical and Biological Products	20170522
LY294002	Sigma-Aldrich	L9908
PD98059	Sigma-Aldrich	P215
SB203580	Sigma-Aldrich	S8307
SP600125	Sigma-Aldrich	S5567
TUNEL Andy Fluor™ 488 Apoptosis Detection Kit	Roche	A050

Supplement Table 3: Primer list

Primer	Forward	Revers	Species
**18s**	TTCCGATAACGAACGAGACTCT	TGGCTGAACGCCACTTGTC	Mouse
**SMA**	GTCCCAGACATCAGGGAGTAA	TCGGATACTTCAGCGTCAGGA	Mouse
**Calponin**	TCTGCACATTTTAACCGAGGTC	GCCAGCTTGTTCTTTACTTCAGC	Mouse
**SM22α**	CAACAAGGGTCCATCCTACGG	ATCTGGGCGGCCTACATCA	Mouse
**Myocardin**	AAGGTCCATTCCAACTGCTC	CCATCTCTACTGCTGTCATCC	Mouse
**KLF4**	CTTTCCTGCCAGACCAGATG	GGTTTCTCGCCTGTGTGAGT	Mouse
